# Spatiotemporal Changes in Fine Particulate Matter Pollution and the Associated Mortality Burden in China between 2015 and 2016

**DOI:** 10.3390/ijerph14111321

**Published:** 2017-10-30

**Authors:** Luwei Feng, Bo Ye, Huan Feng, Fu Ren, Shichun Huang, Xiaotong Zhang, Yunquan Zhang, Qingyun Du, Lu Ma

**Affiliations:** 1 School of Resources and Environmental Science, Wuhan University, Wuhan 430079, China; lwfeng@whu.edu.cn (L.F.); renfu@whu.edu.cn (F.R.); 2 Department of Epidemiology and Biostatistics, School of Health Sciences, Wuhan University, Wuhan 430071, China; yeb@whu.edu.cn (B.Y.); 2017203050003@whu.edu.cn (H.F.); 2017283050055@whu.edu.cn (S.H.); XiaotongZhang@whu.edu.cn (X.Z.); Yun-quanZhang@whu.edu.cn (Y.Z.); 3 Key Laboratory of GIS, Ministry of Education, Wuhan University, Wuhan 430079, China; 4 Key Laboratory of Digital Mapping and Land Information Application Engineering, National Administration of Surveying, Mapping and Geoinformation, Wuhan University, Wuhan 430079, China; 5 Collaborative Innovation Center of Geospatial Technology, Wuhan University, Wuhan 430079, China; 6 Global Health Institute, Wuhan University, 8 Donghunan Road, Wuhan 430072, China

**Keywords:** PM_2.5_, spatiotemporal characteristics, population exposure, mortality burden, China

## Abstract

In recent years, research on the spatiotemporal distribution and health effects of fine particulate matter (PM_2.5_) has been conducted in China. However, the limitations of different research scopes and methods have led to low comparability between regions regarding the mortality burden of PM_2.5_. A kriging model was used to simulate the distribution of PM_2.5_ in 2015 and 2016. Relative risk (RR) at a specified PM_2.5_ exposure concentration was estimated with an integrated exposure–response (IER) model for different causes of mortality: lung cancer (LC), ischaemic heart disease (IHD), cerebrovascular disease (stroke) and chronic obstructive pulmonary disease (COPD). The population attributable fraction (PAF) was adopted to estimate deaths attributed to PM_2.5_. 72.02% of cities experienced decreases in PM_2.5_ from 2015 to 2016. Due to the overall decrease in the PM_2.5_ concentration, the total number of deaths decreased by approximately 10,658 per million in 336 cities, including a decrease of 1400, 1836, 6312 and 1110 caused by LC, IHD, stroke and COPD, respectively. Our results suggest that the overall PM_2.5_ concentration and PM_2.5_-related deaths exhibited decreasing trends in China, although air quality in local areas has deteriorated. To improve air pollution control strategies, regional PM_2.5_ concentrations and trends should be fully considered.

## 1. Introduction

Fine particulate matter (PM_2.5_) is well known for its negative impacts on human health. Notably, it has contributed to 4.24 million deaths, according to the Global Burden of Disease study 2015 (GBD 2015) [1]. Exposure to PM_2.5_ could lead to cardiovascular [2,3,4] and respiratory diseases [5,6], which have been extensively investigated in a number of epidemiological cohort studies. Although the mechanisms are not fully understood, exposure to PM_2.5_ has been found to be linked to certain pregnancy outcomes [7,8] and disorders of the nervous system. Among the diseases associated with PM_2.5_ concentrations, stroke, ischaemic heart disease (IHD), lung cancer (LC) and chronic obstructive pulmonary disease (COPD) ranked as the 1st, 2nd, 4th and 5th highest causes of death in China, as reported in GBD 2015 [1]. Diseases, and even death, can result in various economic problems. According to the World Bank, the cost of the excess deaths associated with air pollution is approximately 1.2% (using the adjusted human capital approach) or 3.8% (using the “value of a statistical life” method) of the GDP [9].

In the past several decades, China has achieved rapid economic growth, industrialization and urbanization, with annual increases in GDP of 9.7% from 1979 to 2015, according to the Chinese Statistical Yearbook 2016, published by China Statistics Press [10]. During the same period, advances in technology and education, coupled with pollution control policies, have had positive effects on decreasing air pollution [11,12,13,14]. Still, cities in China rank among the most polluted in the world, including some international cosmopolitan cities such as Beijing [13,15] and Shanghai [16]. Notably, the North China Plain has experienced poor air conditions and persistent fog and haze [17,18]. An increasing concern over the adverse influences of particulate matter, especially PM_2.5_, on human health could be seen along with the promotion of health awareness among citizens. Some previous studies examined the association between PM_2.5_ and daily mortality in several Chinese cities, including Shanghai [19], Guangzhou [20], Xi’an [21] and Shenyang [22]. However, the cities often explored by researchers were economically advanced or heavily polluted, and low comparability existed between the results due to the various research methods adopted. The methods such as the linear model and the logarithm model have some differences, especially in estimating the air pollution burden at a high polluted level. The linear model produces a linear increase in relative risk (RR) from the lowest air pollution level to the highest (below 50 μg/m^3^). Meanwhile, the marginal increase of logarithm model is more gradual than that of the linear model at higher concentrations. What they have in common is that they both fail to cover the global range of PM_2.5_ exposure and will lead to a deviation at higher PM_2.5_ concentrations [23]. As a result, estimating the health impact of PM_2.5_ in all cities through a more unified and accurate approach is necessary in order for local governments to formulate relevant policies.

The Chinese government started organizing daily reports and forecasts of urban air quality in 2000, but PM_2.5_ was not included in the routine monitoring items until the release of the “Ambient Air Quality Standard” (AAQS) (GB 3095-2012) in 2012. Additionally, at the end of the same year, 74 key cities published hourly PM_2.5_ monitoring data. In 2015, 336 prefecture-level cities adopted a new standard of air quality monitoring and began to monitor various pollutants, including PM_2.5_. Meanwhile, the exploration of spatial and temporal variability in PM_2.5_ has expanded to a national scale, as the coverage of air quality monitoring data sets has expanded and data have become readily available. The air quality monitoring network in China also provides a great opportunity for researchers to conduct PM_2.5_-related research on a national scale.

This study is motivated by multiple factors. It is crucial for policy designers to understand the health benefits of PM_2.5_ control and the health risks arising from the deterioration of air quality. In addition, though there have been some nationwide studies exploring the association between the number of deaths and exposure to PM_2.5_ concentrated in China, more research has focused on certain big cities. In this paper, we used hourly air quality monitoring data in 336 prefecture level cities to explore the spatial and temporal distribution of PM_2.5_ in China and to estimate the numbers and trends of stroke deaths, IHD deaths, LC deaths and COPD deaths over the past two years due to the impact of PM_2.5_ in each city. These deaths are a direct reflection of the health burden of PM_2.5_. Additionally, the ground-level PM_2.5_ data adopted in this study have covered all the cities in China and have high temporal resolutions (as they have been published hourly).

## 2. Materials and Methods

### 2.1. Materials

#### 2.1.1. Ground Monitoring PM_2.5_ Data

**Figure 1:**
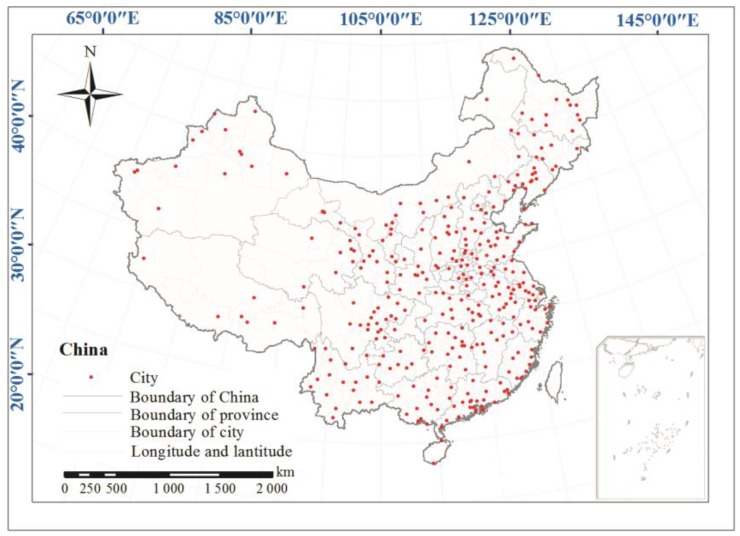
Locations of the 336 Chinese cities with available and sufficient data.

The hourly PM_2.5_ concentrations from January 2015 to December 2016 in 336 cities were obtained from the China National Environmental Monitoring Center (CNEMC), and spatial coverage of the data is illustrated in Figure 1. The micro oscillating balance method and the beta absorption method were used to measure PM_2.5_. The instruments which measured PM_2.5_ concentration in each site were tested by using at least 3 samplers based on HJ 618-2011, according to the regulations published by the Ministry of Environmental Protection of the People’s Republic of China [24]. The CNEMC website [25] is a public website that provides air quality data free of charge, and the data are from national air quality automatic monitoring sites and were initially reviewed by the release system. In this paper, daily, monthly and yearly PM_2.5_ concentrations were all calculated based on hourly data. Although the source and acquisition process were credible, further filtering and correction were necessary because of some routine maintenance activities, communication failures and power outages at monitoring sites could lead to the absence of data. The data were transformed into z scores (standard scores), and data were removed when the following conditions were met: (1) less than 12 h of valid data were available for a day; (2) the absolute z score was larger than 4; or (3) the increase from the previous value was larger than 6 [26]. After the screening and deletion processes, more than 85% of the hourly data could be used for further calculation.

#### 2.1.2. Population and Mortality Data

The population and deaths reported at each monitoring site were collected, summarized and combined in the China Death Monitoring Data Set. The China Death Monitoring Data Set was edited by two departments, the National Health and Family Planning Commission of the People’s Republic of China and the Chinese Center for Disease Control and prevention. The data quality at each monitoring site was evaluated before data collection, and sites with serious missing reports which might affect the overall results were excluded, as described in the dataset instructions. The latest data of the number and cause of deaths were provided by the China Death Monitoring Data Set 2014. This data set included the deaths from LC (C33–C34), IHD (I20–I25), stroke (I60–I69), and COPD (J40–J44), as well as the total number of deaths in the eastern region (including Beijing, Tianjin, Hebei, Liaoning, Shanghai, Jiangsu, Zhejiang, Fujian, Shandong, Guangdong and Hainan), central region (including Shanxi, Jilin, Heilongjiang, Anhui, Jiangxi, and Henan) and western region (including Inner Mongolia, Guangxi, Chongqing, Sichuan, Guizhou, Yunnan, Tibet, Shaanxi, Gansu, Qinghai, Ningxia and Xinjiang) of China. Provincial population and mortality information was obtained from the provincial Statistical Bulletin of National Economic and Social Development.

### 2.2. Methods

#### 2.2.1. Kriging Model

To visualize the regional profiles of PM_2.5_ across China, a kriging model was applied to generate a nationally continuous surface based on the PM_2.5_ concentrations in 336 cities. Kriging is a high-level statistical process used to generate an estimated surface based on a set of scatter points with z values. As a famous interpolation method, it was first used in the mineral industry and later used in a wide variety of disciplines, such as air pollution mapping [27]. Among the various versions of kriging, we employed ordinary kriging, a commonly used variant of the kriging algorithm [28,29], to construct an unbiased estimator without the requirement of a stationary mean of observed values in advance. The predictive formula of the kriging model is shown in Equation (1):

(1) $$Z\left(S_{0}\right)=\sum_{i=1}^{N}\lambda_{i}Z(S_{i})$$

where Z(S_0_) is the predictive value, Z(S_i_) is the measurement at position i, N is the number of measurements, and λ_i_ is the unknown weight of the predictive value at position i. Here, weight relies on not only distance and the predictive positions of measurement points, but also on the spatial arrangement of measurement points. Therefore, the estimation of a semivariogram γ(h) at a distance of h is necessary to provide spatial autocorrelation information for the data set before creating predictive surfaces. To ensure that kriging predictions have positive kriging variances, it is necessary to fit a model—that is, a continuous function or curve—to the empirical semivariogram. Linear, circular, spherical, exponential and Gaussian are some models used to fit different types of phenomena [30,31]. For example, spherical model shows a progressive decrease of spatial autocorrelation until some distance, beyond which the autocorrelation is zero. The formula is shown in Equation (2):

(2) $$\begin{array}{c} \gamma \left(h\right)=C_{0}+C_{1}\left[\frac{3h}{2a}-\frac{1}{2}{\left(\frac{h}{a}\right)}^{3}\right],\;0\leq h\leq a\\ \gamma \left(h\right)=C_{0}+C_{1},\;h>a \end{array}$$

where C_0_, C_1_, C_0_ + C_1_ and a represent nugget, partial sill, sill and range respectively.

#### 2.2.2. Integrated Exposure–Response (IER) Model

An increase in the PM_2.5_ concentration could lead to higher rates of LC, IHD, stroke and COPD. To estimate the number of premature deaths caused by PM_2.5_ in 2015 and 2016, we applied the IER model proposed by Burnett et al. The IER model is an effective predictor of relative risk. RR is the ratio of the probability of an event occurring in an exposed group to the probability of the event occurring in a comparable, non-exposed group. Notably, the IER model combines the RRs of ambient air pollution, second-hand tobacco smoke, household solid cooking fuel and active smoking, and yields reasonable predictions over a range of concentrations that prevail in China and other highly polluted areas. RR can be calculated using Equation (3):

(3) $$\begin{array}{c} {\mathrm{RR}}_{\mathrm{IER}}\left(z\right)=1,\;z<z_{\mathrm{cf}}\\ {\mathrm{RR}}_{\mathrm{IER}}\left(z\right)=1+\alpha \left\{1-\exp\left[-\gamma {\left(z-z_{\mathrm{cf}}\right)}^{\delta }\right]\right\},\;z\geq z_{\mathrm{cf}} \end{array}$$

where z is the exposure to PM_2.5_ and z_cf_ is the PM_2.5_ concentration below which no additional risk exists. Here, we define z_cf_ as a uniform random variable between 5.8 μg/m^3^—the minimum concentration observed in the American Cancer Society Cancer Prevention II cohort [32]—and 8.8 μg/m^3^—the 5th percentile value. For very large z values, RR_IER_ approximates 1 + α. Parameter δ is a power of PM_2.5_ to predict risk over a very large range of concentrations. Further, RR_IER_ (z_cf_ + 1) approximates 1 + αγ. Thus, γ = [RR_IER_ (z_cf_ + 1) − 1]/[RR_IER_ (∞) − 1] can be interpreted as the ratio of the RR at low-to-high exposures. α, γ, and δ are unknown parameters, estimated using nonlinear regression methods according to the RR information and the variance estimates of the logarithms of RRs at different PM_2.5_ concentration from the available literature [33]. The detailed information about the estimates of (α, γ, δ), and the confidence interval for RR_IER_ can be found in the supplemental material.

#### 2.2.3. Estimates of Health Impacts and Mortality Due to Exposure to PM_2.5_

Two assumptions used in this study should be noted before further analysis: (1) that the proportion of deaths caused by four diseases (LC, IHD, stroke and COPD) compared to total deaths was the same in 2015 and 2014 (this assumption was made because the 2015 China Death Monitoring Data Set has not been published as of the writing of this article), and (2) that city-level death rates are the same as the death rate of the associated province (this assumption was made because the death rates are unavailable for many cities).

The deaths impacts (DI) due to PM_2.5_ exposure in each city can be estimated using Equation (4):

(4) $${\mathrm{DI}}_{i,j}={\mathrm{Population}}_{i}\times {\mathrm{Mortality}}_{i,j}\times {\mathrm{PAF}}_{i,j}$$

where DI_i,j_ is the number of deaths due to exposure to PM_2.5_ for disease j in city i. Population_i_ is the exposed population in city i. Mortality_i,j_ represents the mortality of disease j in city i. PAF_i,j_ is the population attributable fraction (PAF) of the cause-specific mortality of disease j in city i. Specifically, PAF can be determined using Equation (5):

(5) $${\mathrm{PAF}}_{i,j}=({\mathrm{RR}}_{\mathrm{Ci},j}-1)/{\mathrm{RR}}_{\mathrm{Ci},j}$$

where RR_Ci,j_ is the RR of the specific mortality of disease j at exposure level C_i_ estimated from the IER model. Non-exposure mortality in each city can be computed based on the exposure mortality and PAF in 2015. Thus, cause-specific mortality in 2016 can easily be determined using Equation (6):

(6) $${\mathrm{PAF}}_{i,j}=({\mathrm{Mortality}}_{i,j}-{\mathrm{Mortality}}_{\mathrm{non}-\mathrm{exposure}})/{\mathrm{Mortality}}_{i,j}$$

Then, with the cause-specific mortality and PAF in 2015 and 2016, the mortality trends associated with changes in the PM_2.5_ concentration per million people in each city can be obtained.

## 3. Results and Discussion

### 3.1. Spatial Distribution of PM_2.5_

**Figure 2:**
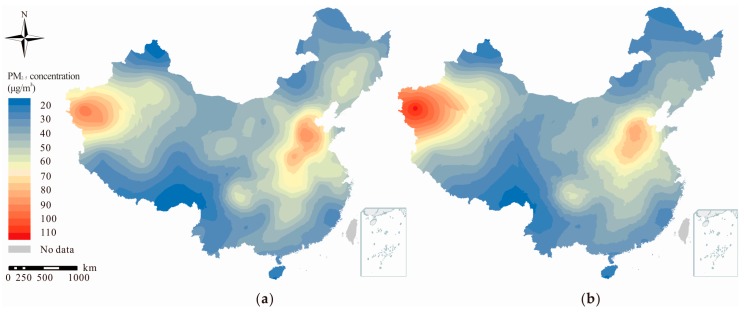
Spatial distribution of the annual fine particulate matter (PM_2.5_) concentration in 2015 (**a**) and 2016 (**b**) across China.

Figure 2 illustrates the spatial distribution of the PM_2.5_ concentration in 2015 and 2016 across China, estimated using the ordinary kriging model performed on the ArcGIS (version 10.3, Environmental Systems Research Institute, Redlands, CA, USA) (selection of some parameters: Spherical model used to fit semivariogram, 1000 set as output cell size and 12 points considered for the search radius). A high degree of similarity can be observed between the PM_2.5_ concentration distribution in 2015 and that in 2014 [34,35]. Specifically, concentrations are high on the North China Plain and low in Southwest China. However, some significant differences can be observed between the spatial patterns of PM_2.5_ in 2015 and 2016, including a shift in the highest PM_2.5_ concentration from the North China Plain to the westernmost part of China. The air quality in the North China Plain area benefited from the Air Pollution Prevention and Control (APPC) action plan initiated in 2013 and the unified classification standard for the early warning of heavy pollution implemented in the Beijing–Tianjin–Hebei region in 2016 [36]. However, emissions caused by the increase in the number of motor vehicles and boilers, coupled with the influence of meteorological factors (increased regional sand weather, and rise of temperature and decrease of precipitation in the winter of 2015/2016) contributed to the aggravated PM_2.5_ pollution in the west of Xinjiang Province [37].

Overall, 72.02% of cities observed a decrease in the PM_2.5_ concentration from 2015 to 2016, among which Hohhot and Xiaogan experienced the most significant decreases (of more than 20 μg/m^3^). The PM_2.5_ concentration in 24.40% of cities increased by less than 10 μg/m^3^. In addition, 7 and 5 cities exhibited an increase in PM_2.5_ concentrations 10–20 μg/m^3^ and over 20 μg/m^3^, respectively. At the provincial level, 23 out of 31 provinces exhibited decreased PM_2.5_ concentrations, and significant decreases were observed in Jilin and Hubei (Table S1). By contrast, Xinjiang exhibited the most obvious increase in its PM_2.5_ concentration among the remaining 8 provinces. All cities in Jilin, Hubei and Shandong exhibited declines in their PM_2.5_ concentrations, and PM_2.5_ in more than 90% of the cities in Heilongjiang, Inner Mongolia, Liaoning, Hunan and Zhejiang decreased. By contrast, the PM_2.5_ concentration in approximately 90.91% of the cities in Shanxi increased to some extent.

According to the WHO air quality guidelines (AQGs), an annual average concentration of 10 μg/m^3^ is set as the long-term guideline value for PM_2.5_, over which significant effects on survival are observed. Besides this guideline, three interim targets are also defined and may benefit countries in gauging progress over time in the process of reducing PM_2.5_ exposures. An annual mean PM_2.5_ concentration of 35 μg/m^3^ is defined as the IT-1 level, which is associated with about a 15% higher long-term mortality risk. The IT-2 level is set at 25 μg/m^3^, and this level lowers the risk of premature mortality by about 6%, relative to the IT-1 level. The IT-3 level is 15 μg/m^3^, reducing the mortality risk by approximately 6% relative to IT-2 level. The proportions of the hourly PM_2.5_ concentrations exceeding the WHO annual threshold values in 2015 and 2016 were not equally distributed at a national scale. Figure S1 shows the distribution of the hourly PM_2.5_ concentrations of all 336 cities in the ranges of ≤10 μg/m^3^, 10–15 μg/m^3^, 15–25 μg/m^3^, 25–35 μg/m^3^ and >35 μg/m^3^. An obvious geographical difference in the distribution of the PM_2.5_ concentration can be observed in Figure S1. The concentration of PM_2.5_ has declined in recent years, but it is still far from the WHO standard. A previous study indicated that the areal proportion of China with a PM_2.5_ concentration of less than 35 μg/m^3^ decreased from 1999 to 2011 [38]. Additionally, more than 50% of cities were in a stage of “non-attainment” in September, although September was the month when the largest number of cities exhibited minimum PM_2.5_ concentrations among 190 Chinese cities in 2014 [34]. Most monitoring sites exceeded the WHO standard, and only 0.38%, 0.41% and 12.93% of stations met the WHO IT-3, IT-2 and IT-1 thresholds, respectively, in China in 2015. Only 1% of susceptible people (of age 61 and over, or 13 and under) lived in areas with relatively safe levels of PM_2.5_ (less than 10 μg/m^3^) in 2010 [39], and approximately 14% (181.08 million) of people were exposed to PM_2.5_ concentrations lower than the WHO IT-1 threshold in 2015 [40].

### 3.2. Temporal Trends of PM_2.5_

**Figure 3:**
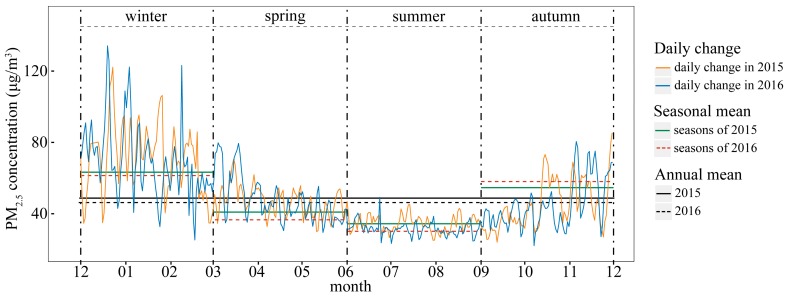
Variation in the PM_2.5_ concentration in 2015 and 2016.

The PM_2.5_ concentration in 2016 was 2.27 μg/m^3^ lower than that of 2015, which is consistent with the decreasing trend in PM_2.5_ from 2014 to 2016 reported in a previous study [41]. Winter, spring, and summer experienced decreases in the PM_2.5_ concentration from 2015 to 2016, especially in spring, with a 4.15 μg/m^3^ decline. The PM_2.5_ concentration exhibited clear seasonal variations and the basic trends were similar in 2015 and 2016. High concentrations in autumn and winter exhibited considerable fluctuations, while low concentrations in spring and summer exhibited moderate fluctuations, as shown in Figure 3. The seasonal variations in air pollution were consistent between years [42]. Severe air pollution occurred in winter months because of high emissions from heating and unfavorable meteorological conditions for pollution dispersion (stagnant weather and temperature inversion) [43]. Crop residue burning can also induce an evident PM_2.5_ increase in winter and autumn [31]. In contrast, sufficient precipitation and active atmospheric circulation led to low pollution on summer days [34,44,45]. The two curves illustrating the change in the PM_2.5_ concentration over the two years differ and display different peak and valley trends and fluctuation ranges.

Diurnal variations in the PM_2.5_ concentration in the four seasons in 2015 and 2016 exhibited similar trends. Notably, after midnight, the concentration gradually increased to the highest value of the day before decreasing to a minimum value and then rising again, as shown in Figure S2. However, the peak and valley times of PM_2.5_ concentrations over the four seasons were not entirely consistent. The maximum difference between the highest and lowest values occurred in winter, reaching 17.72 μg/m^3^ in both years. Meanwhile, the minimum difference occurred in summer, with a value of approximately 9.69 μg/m^3^ in 2015 and 8.36 μg/m^3^ in 2016. Similar trends in the diurnal variation of PM_2.5_ can be observed in some studies conducted by other researchers [18,40,41]. Enhanced anthropogenic activity during rush hour contributed to the morning peak in the PM_2.5_ concentration, which was studied through the comparison between PM_2.5_ diurnal changes in weekdays and weekends [46], as well as the contract between PM_2.5_ concentration diurnal trends in urban and rural areas [47]. Following the morning peak, the decrease in traffic pollution combined with the increase in convective movement led to the decline in the PM_2.5_ concentration. In the evening, traffic pollution and cooking emissions led to a gradual elevation of PM_2.5_ concentration and around midnight, electricity generation caused industrial pollution, increasing the PM_2.5_ concentration. Thus, seasonal shifts in peak and valley times can potentially be attributed to seasonal changes of residents’ behaviors. However, not all cities exhibited diurnal variations that are consistent with the average condition of the 336 cities, which may be explained by the variety of urbanization level, sources of pollution and people’s lifestyle. For example, PM_2.5_ concentration peaks appeared at 8:00 and 20:00 and valleys occurred at 1:00 and 15:00 in Guilin, Guangxi Province. In addition, Changde in Hunan exhibited four small peaks during a day but displayed a narrow range of fluctuation [42].

### 3.3. Disease Burden of PM_2.5_

**Figure 4:**
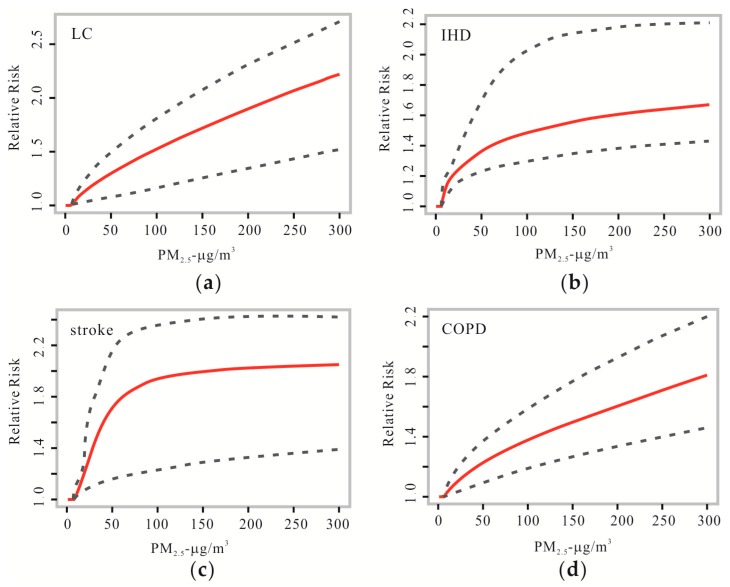
Relationships between the relative risk (RR) of (**a**) lung cancer (LC), (**b**) ischaemic heart disease (IHD), (**c**) stroke, and (**d**) chronic obstructive pulmonary disease (COPD) and the PM_2.5_ concentration predicted by the IER model. Red lines and dotted lines represent predicted values of IER model and 95% CIs respectively.

The PM_2.5_ concentration, RR and standard error were obtained from the aggregated information in the supplemental material of Burnett et al.’s paper [33] for the burdens of diseases (including LC, IHD, stroke and COPD), based on published sources. The relationship between the PM_2.5_ concentration and RR was calculated using the IER model, as shown in Figure 4. The curves of LC and COPD are almost straight lines, which suggest that RR increased uniformly as the PM_2.5_ concentration increased. For the curves corresponding to stroke and IHD, RR increased rapidly when the PM_2.5_ concentration was below 50 μg/m^3^ and then increased slowly. Deaths attributed to PM_2.5_ based on the actual populations in 31 provinces and deaths per million residents in 336 cities were calculated based on the PAF.

**Figure 5:**
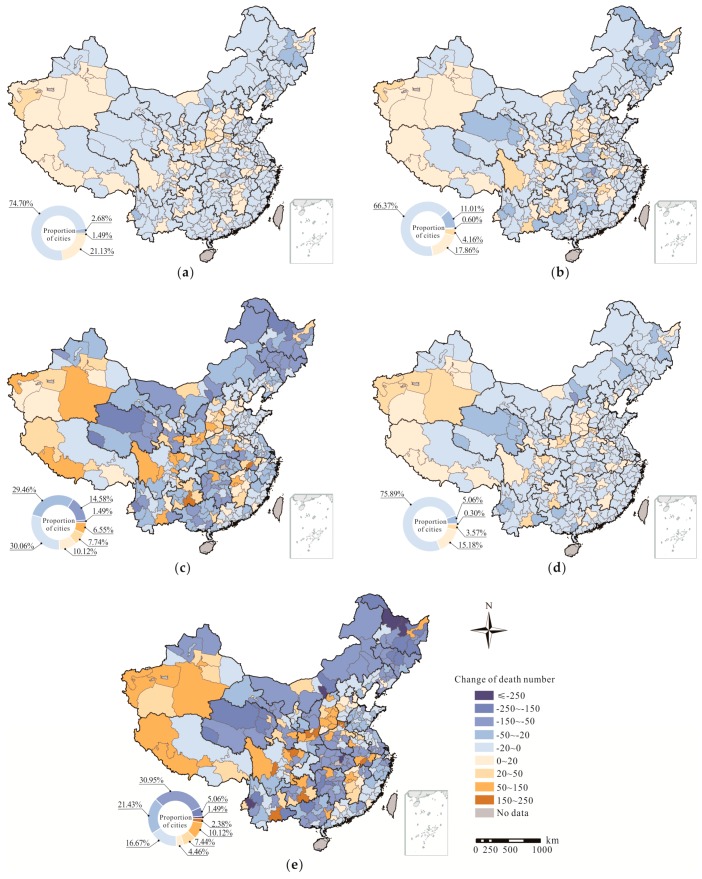
Distributions of changes in (**a**) LC deaths, (**b**) IHD deaths, (**c**) stroke deaths, (**d**) COPD deaths and (**e**) total deaths from 2015 to 2016.

Among 31 provinces, Henan exhibited the largest RRs for four causes of premature mortality, with values of 1.42, 1.44, 1.87 and 1.31 for LC, IHD, stroke and COPD, respectively. In addition, Hainan exhibited the smallest RRs of 1.12, 1.20, 1.20 and 1.10 for LC, IHD, stroke and COPD, respectively. The change in PM_2.5_-related deaths in each province from 2015 to 2016 is listed in Table 1. The total number of deaths due to the four diseases was largest in Henan, at more than 130,000, while the lowest total was observed in Tibet, at less than 2000. This finding reflects an obvious difference between provinces. At the provincial level, 21 provinces experienced different declines in deaths due to changes in PM_2.5_. Heilongjiang, Hunan and Hubei were the top three provinces with the most obvious decreases of 6391, 5304 and 4705 deaths, respectively. In the remaining 10 provinces, Sichuan, Shanxi and Shaanxi exhibited the most significant increases from 2015 to 2016, with 2769, 2697 and 2553 more deaths, respectively. The decreases or increases in deaths per million residents related to PM_2.5_ in 336 Chinese cities were calculated, and the results are presented in Table S2 and Figure 5. At the city level, approximately 80% of cities exhibited fewer deaths due to the four diseases. For LC, IHD and COPD, the distributions of changes in deaths were similar, with approximately 70% of the cities exhibiting a decrease in deaths—ranging from 20% to 50%—and 17% of cities exhibiting an increase in deaths—ranging from 0% to 20%. The proportions of cities in different ranges were similar for stroke. In terms of the change in the total number of deaths due to PM_2.5_ from 2015 to 2016, Baoshan, Yichun and Hohhot exhibited the most obvious declines among the 231 cities with fewer deaths, while 23 cities remained unchanged. The remaining 82 cities exhibited more deaths, with Qiannan, Chizhou and Xianyang experiencing the three highest increases.

Our study indicated that approximately 1,126,000 deaths were caused by PM_2.5_ in 2015 across China. This total is slightly larger than the value estimated by GBD 2015 (1,108,000) [48]. Moreover, our estimate decreased to approximately 1,092,000 in 2016 due to the overall decline in the PM_2.5_ concentration. Notably, the numbers of deaths due to LC, IHD, stroke and COPD caused by PM_2.5_ totalled 130,000, 284,000, 592,000 and 120,000, respectively, in 2015 and 124,000, 278,000, 573,000 and 117,000, respectively, in 2016. Our estimates are comparable with those of previous studies. These studies found that premature deaths in China caused by PM_2.5_ totaled 807,000 in 2004, 1,250,000 in 2012 [49], 1,367,000 in 2013 [50], and 1,600,000 in 2014 [51]. In 2015, the number of deaths per million people attributed to PM_2.5_ was 813 in China. This total was more than four times that in the United States and Japan. Additionally, China accounted for approximately a quarter of global deaths (4,241,000), which suggests that air pollution remains a serious problem in China. However, according to our research, air pollution in China improved significantly from 2015 to 2016, based on the fact that 72.02% of the cities exhibited varying decreases in their PM_2.5_ concentrations, and 68.75% of the cities exhibited decreases in mortality attributed to PM_2.5_. Improvements in air quality in China indicate that the policies and measures taken by the government have achieved initial success. To ensure the continuity and effectiveness of air pollution control, it is necessary to reduce heating, cooking and agricultural emissions [52] and adjust regional policies according to changes in PM_2.5_ concentration.

### 3.4. Strengths and Limitations

Few studies have explored the PM_2.5_-related mortality burden using local health data (including population and mortality data) in China, and most studies have focused on key cities or regions instead of the entire country. Due to the adoption of different algorithms, low comparability exists between these studies. Our study used hourly, ground-level PM_2.5_ monitoring data, population data and mortality data to estimate the deaths in China caused by PM_2.5_ using the IER model and PAF. This approach overcomes the limitations of small-scale research. Moreover, these findings can be used to inform people regarding the health impacts of PM_2.5_ pollution and provide guidance for regional policy design.

However, some limitations and uncertainties exist in our study. The evaluation was based on city-level PM_2.5_ concentrations calculated by averaging the data at all sites, which is the common method used to report daily air quality to the public. However, the uneven distribution of monitoring stations, including more in urban areas and less in suburban and rural areas, makes a simple averaging method less accurate than more complex methods [40]. In addition, the assumptions proposed before further calculations were sources of uncertainties as well, but the assumptions were inevitable due to the unavailability of certain data. Furthermore, the IER model was adopted to estimate the relationships between PM_2.5_ and the RRs of four diseases in our study. However, a highly accurate exposure-response model based completely on Chinese cohort studies should be developed in the future, even though the IER model provides reasonable predictions in China and other heavily polluted areas [49].

## 4. Conclusions

The spatial distributions, temporal trends and health burdens of PM_2.5_ in 336 Chinese cities in 2015 and 2016 were evaluated based on hourly PM_2.5_ concentrations. The annual average PM_2.5_ concentration decreased by 2.27 μg/m^3^, from 48.33 μg/m^3^ in 2015 to 46.06 μg/m^3^ in 2016. Additionally, the region with the highest PM_2.5_ concentration shifted from the North China Plain in 2015 to western Xinjiang Province in 2016. Although the PM_2.5_ levels in China are still far above the WHO standard, 70.02% of Chinese cities exhibited decreases in PM_2.5_ concentrations between 2015 and 2016. Moreover, 34,164 deaths (including 6102 LC deaths, 5701 IHD deaths, 19,193 stroke deaths and 3168 COPD deaths) were avoided due to the overall decrease in the PM_2.5_ concentration in China from 2015 to 2016. New findings in this paper could enhance public awareness regarding the health risks caused by PM_2.5_. We urge governments in Chinese provinces and cities to take effective measures to curb air pollution. In addition, to avoid a rebound in PM_2.5_ pollution, sustainable strategies and joint actions among cities should be considered.

## Figures and Tables

**Table 1:** Changes in deaths due to exposure to PM_2.5_ at the provincial level.

Province	Population (×10^6^)		Attributable Deaths in 2015 (×10^3^)					Attributable Deaths in 2016 (×^3^)					Change from 2015 to 2016 ^a^				
	2015−2016		LC	IHD	Stroke	COPD	SUM	LC	IHD	Stroke	COPD	SUM	LC	IHD	Stroke	COPD	SUM
Heilongjiang	38.12	37.99	3.18	11.20	21.59	3.21	39.17	2.64	10.11	17.36	2.66	32.78	−539	−1084	−4225	−543	−6391
Hunan	67.83	68.22	7.19	23.07	46.41	7.27	83.94	6.54	21.95	43.48	6.68	78.64	−659	−1122	−2930	−594	−5304
Hubei	58.52	58.85	6.02	17.89	36.08	6.06	66.06	5.24	17.10	33.60	5.42	61.35	−784	−797	−2481	−643	−4705
Jilin	27.53	27.33	2.35	7.55	15.18	2.38	27.47	1.96	6.68	13.01	2.05	23.70	−394	−866	−2177	−326	−3762
Guangxi	47.96	48.38	3.24	8.77	22.96	6.59	41.56	2.87	8.30	20.81	5.94	37.92	−363	−476	−2155	−642	−3636
Jiangsu	79.76	79.99	10.40	12.86	23.95	4.03	51.23	9.48	12.21	22.46	3.72	47.87	−919	−643	−1493	−312	−3366
Guangdong	108.49	109.99	6.08	9.01	15.34	2.32	32.74	5.58	8.52	13.68	2.21	29.99	−503	−484	−1654	−115	−2756
Yunnan	47.42	47.71	2.57	7.78	18.09	5.28	33.71	2.30	7.52	16.33	4.93	31.08	−272	−254	−1757	−347	−2630
Shandong	98.47	99.47	14.27	16.44	29.82	5.45	65.98	13.36	15.84	29.07	5.13	63.40	−911	−606	−749	−312	−2578
Inner Mongolia	25.11	25.20	1.47	4.07	10.51	2.98	19.03	1.29	3.71	8.97	2.68	16.65	−171	−356	−1544	−304	−2376
Liaoning	43.82	43.78	4.98	6.37	11.87	1.94	25.16	4.64	6.18	10.87	1.77	23.46	−337	−183	−1001	−171	−1691
Henan	94.80	95.32	12.92	37.49	73.49	13.22	137.11	12.68	36.84	73.05	12.86	135.43	−238	−649	−443	−356	−1686
Zhejiang	55.39	55.90	4.98	6.58	11.89	1.90	25.35	4.67	6.26	11.09	1.73	23.75	−313	−319	−800	−165	−1597
Shanghai	24.15	24.20	2.17	2.75	5.07	0.82	10.81	1.89	2.53	4.53	0.72	9.67	−276	−219	−548	−106	−1148
Gansu	26.00	26.10	1.75	4.75	12.45	3.57	22.53	1.62	4.62	11.86	3.40	21.50	−134	−130	−586	−174	−1024
Hebei	74.25	74.70	9.34	10.76	19.52	3.56	43.18	9.17	10.58	19.18	3.47	42.39	−172	−186	−338	−98	−795
Qinghai	5.88	5.93	0.41	1.11	2.89	0.81	5.21	0.35	1.01	2.53	0.73	4.62	−60	−92	−357	−79	−588
Fujian	38.39	38.74	2.67	4.02	6.45	1.03	14.18	2.54	4.06	6.18	0.97	13.75	−125	37	−275	−65	−429
Anhui	61.44	61.96	5.78	18.09	36.68	5.90	66.46	5.65	18.25	36.49	5.70	66.09	−133	153	−190	−198	−369
Hainan	9.11	9.17	0.44	0.76	0.89	0.16	2.25	0.37	0.73	0.72	0.15	1.96	−70	−33	−174	−17	−294
Beijing	21.71	21.73	2.33	2.69	4.88	0.89	10.79	2.28	2.69	4.83	0.86	10.66	−54	3	−51	−29	−132
Ningxia	6.68	6.75	0.36	0.95	2.53	0.71	4.55	0.36	0.96	2.55	0.72	4.60	4	10	27	7	48
Tibet	3.24	3.31	0.13	0.41	0.86	0.25	1.64	0.13	0.41	0.93	0.27	1.75	3	8	71	26	107
Guizhou	35.30	35.55	2.23	6.82	16.29	4.64	29.97	2.24	6.87	16.41	4.67	30.19	16	49	118	33	217
Tianjin	15.47	15.62	1.82	2.14	3.86	0.68	8.50	1.88	2.16	4.00	0.71	8.75	65	21	132	31	249
Chongqing	30.17	30.48	2.82	7.19	19.20	5.67	34.88	2.85	7.26	19.40	5.73	35.24	30	76	203	60	369
Xinjiang	23.60	23.98	1.38	3.53	9.42	2.78	17.11	1.59	3.78	10.36	3.20	18.92	203	251	939	414	1807
Jiangxi	45.66	45.92	3.84	12.98	25.51	3.80	46.13	4.01	13.45	26.92	4.02	48.41	171	471	1411	223	2277
Shaanxi	37.93	38.13	3.02	7.74	20.75	6.01	37.51	3.23	8.21	22.03	6.59	40.06	218	472	1283	580	2553
Shanxi	36.64	36.82	3.23	10.10	20.48	3.29	37.10	3.55	10.70	21.97	3.59	39.80	320	597	1489	292	2697
Sichuan	82.04	82.62	6.64	17.72	47.06	13.27	84.69	6.94	18.37	48.12	14.03	87.46	295	650	1062	762	2769
SUM	1371	1380	130	284	592	120	1126	124	278	573	117	1092	−6102	−5701	−19,193	−3168	−34,164

^a^ “−” reflects a decrease in deaths from 2015 to 2016.

## Supplementary Materials

The following are available online at www.mdpi.com/1660-4601/14/11/1321/s1, Estimates of (α, γ, δ), Estimates of confidence interval for RR_IER_, Figure S1: Proportion distributions of hourly PM_2.5_ concentrations in 336 cities. Figure S2: Diurnal variation of PM_2.5_ concentration in four seasons over 2015 and 2016. Table S1: Change of PM_2.5_ concentration in provinces from 2015 to 2016. and Table S2: Changes in deaths caused by fluctuations of PM_2.5_ concentrations from 2015 to 2016 in 336 Chinese cities.
