# Supplementary material for: Spatiotemporal Changes in Fine Particulate Matter Pollution and the Associated Mortality Burden in China between 2015 and 2016
Source: Int J Environ Res Public Health. 2017 Oct 30;14(11):1321. doi: 10.3390/ijerph14111321 (PMC5707960; doi:10.3390/ijerph14111321)
Supplement: Supplementary file 1 [file ijerph-14-01321-s001.pdf]

International Journal of
Environmental Research and Public Health

MDPI

Supplemental Material

Spatiotemporal changes in $PM_{2.5}$ pollution and the associated mortality burden in China between 2015 and 2016

Luwei Feng [1,a], Bo Ye [2,a], Huan Feng [2], FuRen [1,3,4], Shichun Huang [2], Xiaotong Zhang [2], Yunquan Zhang [2], Qingyun Du [1,3,4,5,*] and Lu Ma [2,6,*]

[1] School of Resources and Environmental Science, Wuhan University, 129 Luoyu Road, Wuhan 430079, China; lwfeng@whu.edu.cn (L.F.); renfu@whu.edu.cn (F.R.)
[2] Department of Epidemiology and Biostatistics, School of Health Sciences, Wuhan University, 185 Donghu Road, Wuchang District, Wuhan 430071, China; yeb@whu.edu.cn (B.Y.); 2017203050003@whu.edu.cn (H.F.); 2017283050055@whu.edu.cn (S.H.); 2013302280020@whu.edu.cn (X.Z.); Yun-quanZhang@whu.edu.cn (Y.Z.)
[3] Key Laboratory of GIS, Ministry of Education, Wuhan University, 129 Luoyu Road, Wuhan 430079, China
[4] Key Laboratory of Digital Mapping and Land Information Application Engineering, National Administration of Surveying, Mapping and Geoinformation, Wuhan University, 129 Luoyu Road, Wuhan 430079, China
[5] Collaborative Innovation Center of Geospatial Technology, Wuhan University, 129 Luoyu Road, Wuhan 430079, China
[6] Global Health Institute, Wuhan University, 8 Donghunan Road, Wuchang District, Wuhan 430072, China
* Correspondence: qydu@whu.edu.cn (Q.D.); Tel.: + 86-27-8766-4557 (Q.D.); malu@whu.edu.cn (L.M.); Tel.: +86-27-6875-8815 (L.M.)

Table of Contents	Page
Estimates of (α, γ, δ)	2
Estimates of confidence interval for RR_{IER}	2
Table S1	3
Figure S1	4
Figure S2	5
Table S2	5
Reference	12

Estimates of (α, γ, δ): A set of RR estimates $\{\hat{r}_1^{(s)},\ldots,\hat{r}_{Ks}^{(s)}, s=1,\ldots,S\}$ and corresponding confidence intervals based on $PM_{2.5}$ concentrations $\{z_1^{(s)},\ldots,z_{Ks}^{(s)}, s=1,\ldots,S\}$ can be obtained from previous research, where S means different types of $PM_{2.5}$ sources and Ks is the number of RR estimates available from for source type S. In order to estimate α, δ and γ, flowing steps are needed: First, determine the logarithm of the relative risk estimates $\{\hat{r}_1^{(s)},\ldots,\hat{r}_{Ks}^{(s)}, s=1,\ldots,S\}$ denoted by $\{\hat{\gamma}_1^{(s)},\ldots,\hat{\gamma}_{Ks}^{(s)}, s=1,\ldots,S\}$ and then denote the standard error of $\{\hat{\gamma}_1^{(s)},\ldots,\hat{\gamma}_{Ks}^{(s)}, s=1,\ldots,S\}$ by $\{\hat{v}_1^{(s)},\ldots,\hat{v}_{Ks}^{(s)}, s=1,\ldots,S\}$. After that, generate 1000 realizations of the log-relative risks assuming a normal distribution with mean $\{\hat{\gamma}_1^{(s)},\ldots,\hat{\gamma}_{Ks}^{(s)}, s=1,\ldots,S\}$ and standard deviation $\{\hat{v}_1^{(s)},\ldots,\hat{v}_{Ks}^{(s)}, s=1,\ldots,S\}$ and take their exponents. One thousand estimates of (α, γ, δ) could be obtained for each set of simulated RRs. The estimation routine did not converge in a small percentage of cases (< 5%) due to a set of simulated RR that were not consistent with the risk model form in our practical processing. Thus we continue to simulate relative risks until 1000 estimates of (α, γ, δ) were obtained [1].

Estimates of confidence interval for RR_{IER}: We simulated 1,000 sets of source type–specific RRs based on their point estimates and CIs and fit the IER model to these simulated values, obtaining 1,000 sets of parameter estimates of (α, γ, δ) and z_{cf}. Using these parameters, we then generated

1,000 IER values over the global concentration range. The mean of these 1000 IER values at a specific concentration was used as our central estimate of relative risk, and the 2.5% and 97.5% values among the 1000 IER values were used to form the lower and upper confidence intervals for each disease at each specific concentration [1].

Table S1. Change of $PM_{2.5}$ concentration in provinces from 2015 to 2016.

Province	Average change of $PM_{2.5}$ from 2015 to 2016 per city (μg/m³) [a]	Number of cities [b]	Cities with increasing $PM_{2.5}$		Cities with decreasing $PM_{2.5}$	
			Number	Rate (%)	Number	Rate (%)
Jilin	−9.66	9	0	0	9	100
Hubei	−9.05	13	0	0	13	100
Shanghai [c]	−7.62	1	0	0	1	100
Shandong	−7.05	17	0	0	17	100
Qinghai	−6.81	8	1	12.50	7	87.50
Heilongjiang	−6.50	13	1	7.69	12	92.31
Inner Mongolia	−6.38	12	1	8.33	11	91.67
Jiangsu	−5.84	13	0	0	13	100
Liaoning	−5.51	14	1	7.14	13	92.86
Hunan	−4.89	14	1	7.14	13	92.86
Zhejiang	−4.06	11	1	9.09	10	90.91
Guangxi	−3.32	14	2	14.29	12	85.71
Hainan	−2.24	2	0	0	2	100
Guangdong	−2.15	21	3	14.29	18	85.71
Yunnan	−2.11	16	5	31.25	11	68.75
Hebei	−2.11	11	4	36.36	7	63.64
Henan	−2.05	17	7	41.18	10	58.82
Gansu	−1.92	14	4	28.57	10	71.43
Beijing [c]	−1.11	1	0	0	1	100
Fujian	−0.85	9	2	22.22	7	77.78
Anhui	−0.29	16	6	37.50	10	62.50
Ningxia	−0.25	5	2	40	3	60
Guizhou	−0.04	9	4	44.44	5	55.56
Chongqing [c]	0.14	1	1	100	0	0
Sichuan	0.72	21	12	57.14	9	42.86
Tibet	1.10	7	4	57.14	3	42.86
Jiangxi	1.43	11	8	72.73	3	27.27
Tianjin [c]	2.79	1	1	100	0	0
Shaanxi	4.74	10	5	50	5	50
Shanxi	5.79	11	10	90.91	1	9.09
Xinjiang	7.55	14	8	57.14	6	42.86
**SUM**	**−2.34**	**336**	**94**	**27.98**	**242**	**72.02**

[a] "+" reflects an increase in $PM_{2.5}$ from 2015 to 2016, and "−" reflects a decrease in $PM_{2.5}$ from 2015 to 2016.

[b] Cities without records of $PM_{2.5}$ are not included.

[c] Shanghai, Beijing, Chongqing and Tianjin are municipalities of China.

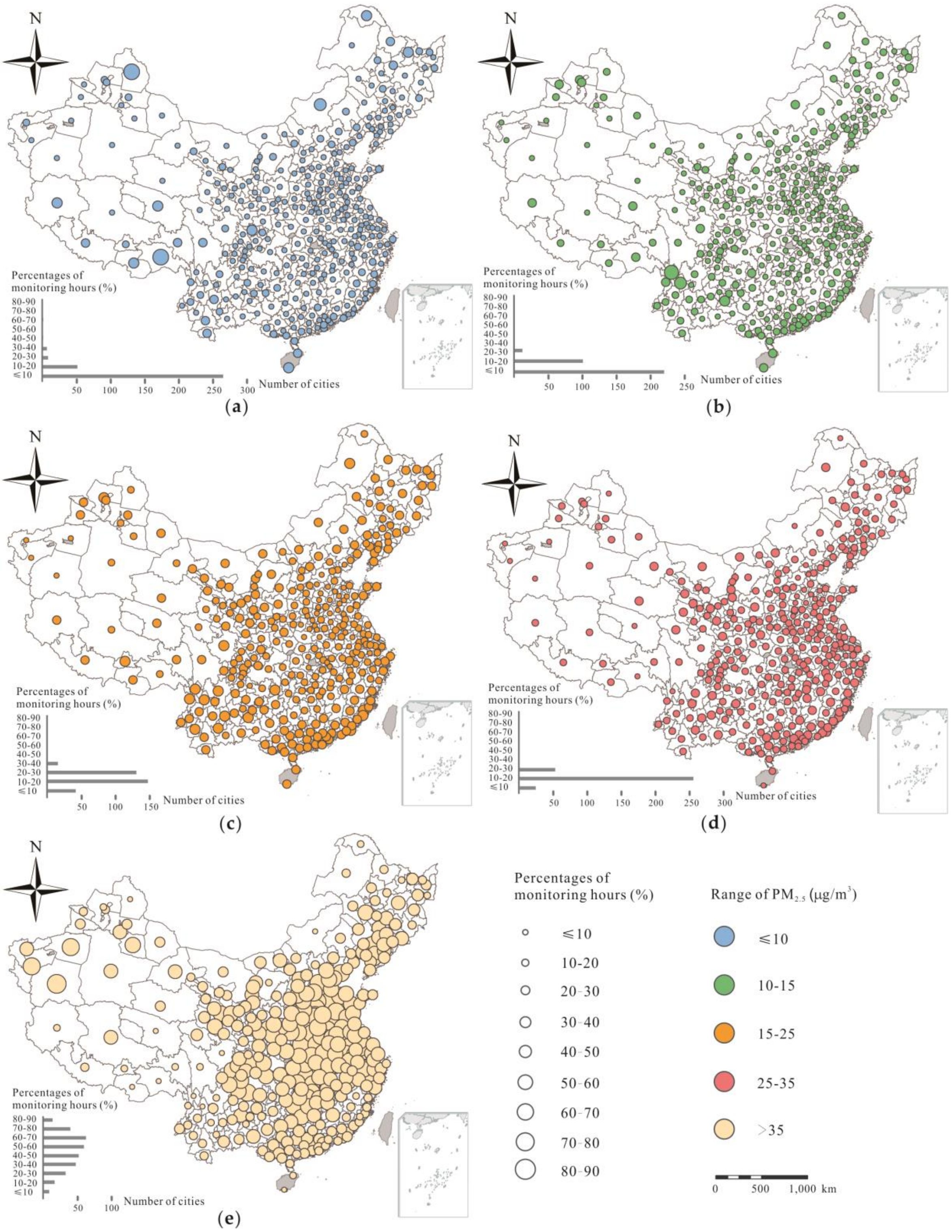

Figure S1. Proportion distributions of hourly $PM_{2.5}$ concentrations (in the range of ≤10 μg/m^3 (**a**), 10–15 μg/m^3 (**b**), 15–25 μg/m^3 (**c**), 25–35 μg/m^3 (**d**), > 35 μg/m^3 (**e**)) in 336 cities.

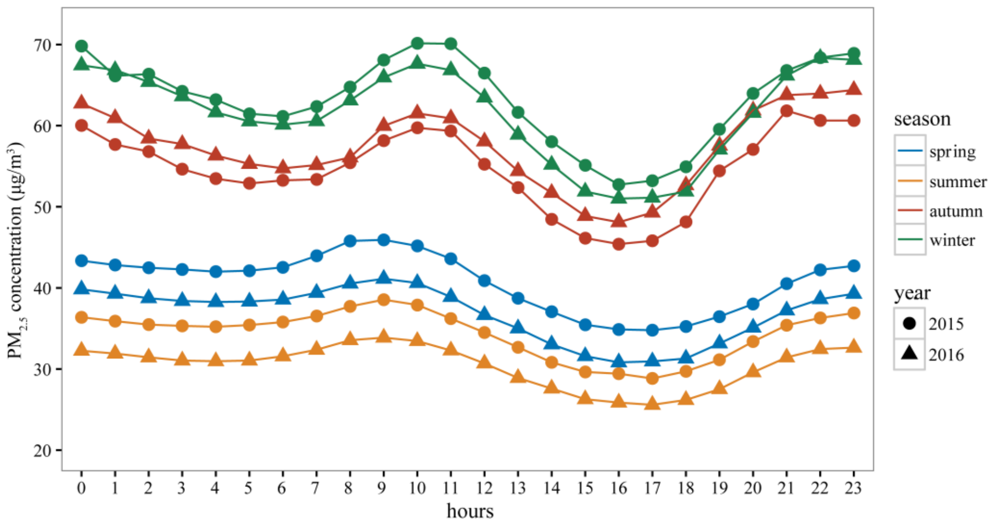

Figure S2. Diurnal variation of $PM_{2.5}$ concentration in four seasons over 2015 and 2016.

Table S2. Changes in deaths caused by fluctuations of $PM_{2.5}$ concentrations from 2015 to 2016 in 336 Chinese cities.

Province	City	$PM_{2.5}$ (μg/m³)		Change of death number from 2015 to 2016				
		2015	2016	LC	IHD	stroke	COPD	sum
**Heilongjiang**	Yichun	26	17	-22.31	-58.16	-219.54	-24.40	-324.41
	Heihe	31	22	-18.12	-47.33	-194.99	-19.18	-279.62
	Mudanjiang	48	37	-20.06	-35.90	-138.45	-22.60	-217.01
	Shuangyashan	42	33	-17.11	-36.44	-125.95	-13.90	-193.41
	Hegang	43	34	-17.11	-27.33	-124.41	-18.38	-187.23
	Harbin	65	51	-22.04	-42.97	-85.71	-17.37	-168.08
	Daxing'anling	25	21	-11.15	-29.08	-105.56	-9.85	-155.64
	Qiqihar	49	41	-13.37	-26.73	-91.76	-13.56	-145.42
	Daqing	44	38	-13.58	-27.13	-76.09	-9.19	-125.99
	Jixi	27	25	-3.69	-9.62	-45.54	-4.88	-63.73
	Suihua	37	34	-7.01	-9.25	-40.52	-4.71	-61.49
	Qitaihe	37	36	-3.51	-9.25	-10.13	0.00	-22.88
	Kiamusze	29	31	3.65	9.54	33.19	4.84	51.22
**Jilin**	Changchun	54	41	-19.16	-29.64	-105.16	-18.63	-172.60
	Yanbian	61	46	-18.74	-36.26	-94.92	-18.34	-168.25
	Liaoyuan	59	46	-16.18	-29.21	-88.11	-14.79	-148.29
	Siping	44	36	-14.23	-30.30	-87.66	-11.55	-143.75
	Jilin	58	46	-16.18	-29.21	-81.23	-14.79	-141.41
	Songyuan	51	42	-11.12	-22.39	-83.59	-15.03	-132.13
	Baicheng	58	48	-13.48	-29.21	-59.07	-11.09	-112.86
	Tonghua	36	31	-8.89	-15.62	-76.88	-7.90	-109.28
	Baishan	52	50	-2.76	-7.41	-15.11	-3.76	-29.04
**Qinghai**	Haixi	38	27	-16.51	-28.89	-157.66	-36.56	-239.63
	Hainan	42	31	-18.81	-28.46	-136.68	-29.01	-212.95
	Haibei	39	31	-13.65	-22.94	-107.18	-21.94	-165.71
	Haidong	57	46	-12.73	-16.46	-80.45	-27.61	-137.24
	Golog	43	37	-8.06	-11.38	-71.47	-21.57	-112.49
	Huangnan	51	45	-7.81	-11.13	-52.68	-21.05	-92.67
	Yushu	19	17	-6.05	-6.31	-43.15	-7.92	-63.42
	Xining	48	50	2.62	5.61	15.32	0.00	23.55
**Hubei**	Xiaogan	71	46	-32.65	-52.76	-141.51	-30.21	-257.14
	Jingmen	69	57	-16.44	-30.15	-52.42	-11.42	-110.43
	Wuhan	68	57	-16.44	-30.15	-45.18	-11.42	-103.19
	Xiangyang	54	47	-11.54	-15.62	-47.51	-11.79	-86.47
	Huangshi	66	57	-13.80	-22.77	-37.85	-7.67	-82.10
	Jingzhou	69	60	-10.96	-22.61	-37.44	-7.61	-78.63
	Suizhou	64	56	-11.12	-15.29	-38.06	-11.51	-75.98
	Ezhou	53	47	-8.72	-15.62	-39.82	-7.92	-72.09
	Shiyan	52	47	-8.72	-15.62	-39.82	-7.92	-72.09
	Huanggang	57	51	-8.59	-15.51	-38.92	-7.79	-70.82

	Enshi	66	59	-11.04	-15.18	-30.28	-7.67	-64.18
	Xianning	71	64	-8.16	-15.07	-29.79	-7.55	-60.58
	Yichang	68	62	-8.22	-15.07	-22.59	-7.61	-53.50
**Inner Mongolia**	Hinggan	67	39	-29.61	-41.66	-158.58	-58.13	-287.98
	Ulanqab	45	33	-13.79	-24.35	-115.00	-24.80	-177.94
	Hohhot	36	29	-9.57	-15.06	-87.54	-19.08	-131.25
	Hulunbuir	51	42	-8.98	-14.40	-71.38	-24.20	-118.96
	Tongliao	54	47	-8.85	-9.53	-38.49	-18.00	-74.86
	Alxa	36	32	-4.79	-5.02	-51.06	-12.72	-73.59
	Erdos	27	24	-4.99	-10.36	-48.88	-6.58	-70.82
	Wuhai	17	15	-2.65	-10.97	-38.49	-6.89	-59.00
	Chifeng	40	37	-4.67	-4.94	-35.10	-12.50	-57.22
	Xilingol	36	33	-2.39	-5.02	-36.47	-6.36	-50.25
	Bayan Nur	48	47	-2.26	0.00	-6.60	-6.10	-14.97
	Baotou	40	43	2.34	4.94	28.08	6.25	41.61
**Hunan**	Hengyang	51	41	-17.24	-27.78	-113.12	-18.64	-176.78
	Huaihua	54	44	-16.98	-27.58	-93.18	-18.49	-156.23
	Yiyang	53	44	-13.69	-27.58	-84.35	-13.98	-139.59
	Changsha	46	40	-10.51	-27.99	-68.37	-9.47	-116.34
	Xiangtan	44	39	-10.59	-18.80	-69.20	-9.55	-108.14
	Zhangjiajie	60	53	-13.28	-18.12	-54.35	-13.65	-99.40
	Yueyang	49	44	-6.95	-18.52	-57.22	-9.40	-92.09
	Xiangxi	49	45	-6.95	-18.52	-47.69	-9.40	-82.55
	Zhuzhou	54	49	-10.19	-9.19	-37.27	-9.25	-65.90
	Loudi	57	52	-6.74	-9.13	-36.64	-9.17	-61.68
	Shaoyang	57	52	-6.74	-9.13	-36.64	-9.17	-61.68
	Yongzhou	52	49	-6.84	-9.19	-28.12	-4.66	-48.81
	Chenzhou	54	51	-6.79	-9.19	-18.64	-4.62	-39.24
	Changde	47	54	14.01	18.66	57.90	14.21	104.78
**Guangxi**	Hechi	42	32	-16.07	-22.69	-120.21	-28.91	-187.88
	Yulin	38	32	-8.23	-11.52	-82.71	-21.87	-124.33
	Nanning	40	35	-8.10	-11.43	-64.92	-14.46	-98.91
	Guigang	41	37	-5.40	-11.35	-48.39	-14.46	-79.59
	Liuzhou	49	44	-5.23	-11.10	-45.54	-14.10	-75.97
	Hezhou	39	35	-5.44	-11.43	-49.31	-7.29	-73.47
	Fangchenggang	31	28	-2.84	-11.79	-44.80	-7.48	-66.90
	Chongzuo	37	34	-5.49	-5.76	-33.51	-7.35	-52.11
	Laibin	43	40	-2.68	-5.67	-31.66	-7.17	-47.18
	Guilin	47	44	-2.64	-5.59	-30.72	-7.11	-46.05
	Beihai	31	29	-2.84	-5.89	-26.88	-7.48	-43.09
	Baise	43	41	-2.68	0.00	-23.75	-7.17	-33.59
	Qinzhou	36	36	0.00	0.00	0.00	0.00	0.00
	Wuzhou	36	39	5.53	11.61	33.73	7.35	58.22
**Yunnan**	Baoshan	37	22	-26.02	-48.55	-247.18	-46.48	-368.22
	Dali	29	22	-12.05	-25.04	-144.63	-23.85	-205.57
	Wenshan	38	30	-11.56	-24.27	-122.01	-23.04	-180.88
	Xishuangbanna	30	26	-8.96	-12.52	-67.02	-15.76	-104.26
	Kunming	28	26	-6.03	-6.31	-39.12	-7.95	-59.41
	Lincang	29	27	-3.01	-6.26	-38.57	-7.95	-55.79
	Nujiang	22	21	-3.12	-6.46	-21.58	-8.16	-39.33
	Hongde	16	15	0.00	-6.74	-23.85	-8.39	-38.98
	Pu'er	25	24	-3.07	-6.36	-20.44	0.00	-29.87
	Zhaotong	33	32	-2.94	0.00	-18.37	-7.81	-29.12
	Chuxiong	22	22	0.00	0.00	0.00	0.00	0.00
	Lijiang	16	16	0.00	0.00	0.00	0.00	0.00
	Yuxi	24	24	0.00	0.00	0.00	0.00	0.00
	Qujing	30	31	0.00	6.26	19.15	0.00	25.41
	Diqing	31	32	2.99	6.21	18.88	0.00	28.08
	Honghe	35	44	14.57	24.46	117.04	23.24	179.31
**Shanghai**	Shanghai	53	45	-11.57	-9.24	-23.02	-4.44	-48.27
**Hainan**	Sanya	17	13	-12.23	-16.79	-40.63	-3.99	-73.64
	Haikou	21	20	-3.97	-4.09	-9.50	0.00	-17.56
**Jiangsu**	Suqian	56	46	-19.75	-12.72	-39.22	-6.11	-77.80
	Lianyungang	53	44	-16.04	-12.82	-35.91	-6.16	-70.93
	Nantong	56	47	-19.75	-12.72	-31.37	-6.11	-69.96
	Zhenjiang	58	50	-15.68	-12.63	-23.40	-6.06	-57.77

	Taizhou	60	53	-15.57	-8.42	-23.14	-6.01	-53.14
	Nanjing	55	48	-11.94	-12.72	-23.66	-4.07	-52.40
	Yancheng	48	43	-12.22	-8.67	-24.50	-4.14	-49.53
	Changzhou	56	51	-11.85	-8.48	-15.69	-2.04	-38.06
	Wuxi	60	56	-7.78	-4.21	-11.57	-4.01	-27.57
	Suzhou	59	55	-7.84	-4.21	-11.63	-2.02	-25.71
	Yangzhou	54	51	-7.96	-4.27	-7.93	-2.04	-22.20
	Huai'an	57	54	-3.95	-4.24	-11.70	-2.02	-21.91
	Xuzhou	63	61	-3.86	0.00	-7.63	-1.99	-13.48
**Liaoning**	Shenyang	69	53	-28.34	-19.32	-38.68	-9.25	-95.59
	Panjin	50	40	-18.94	-16.14	-45.40	-5.82	-86.30
	Dalian	46	38	-19.24	-16.26	-38.98	-5.87	-80.34
	Liaoyang	57	47	-18.51	-11.93	-32.90	-7.58	-70.92
	Fushun	51	43	-15.15	-12.10	-33.85	-5.77	-66.89
	Benxi	52	45	-15.04	-12.02	-29.92	-5.77	-62.75
	Huludao	52	47	-11.28	-8.01	-18.70	-3.85	-41.84
	Tieling	55	49	-11.19	-7.95	-18.49	-3.82	-41.45
	Dandong	45	42	-3.88	-4.09	-15.68	-1.97	-25.63
	Fuxin	47	44	-3.85	-4.06	-15.40	-1.96	-25.27
	Yingkou	47	45	-3.85	-4.06	-11.55	-1.96	-21.42
	Jinzhou	58	55	-7.35	-3.95	-7.31	-1.89	-20.50
	Chaoyang	40	40	0.00	0.00	0.00	0.00	0.00
	Anshan	50	53	3.79	4.03	7.57	1.94	17.33
**Gansu**	Pingliang	48	42	-7.85	-11.18	-61.08	-21.15	-101.25
	Dingxi	41	36	-8.10	-17.02	-56.45	-14.46	-96.02
	Linxia	47	41	-7.91	-11.18	-61.44	-14.22	-94.74
	Zhangye	41	37	-5.40	-11.35	-48.39	-14.46	-79.59
	Longnan	34	31	-5.58	-11.61	-51.61	-7.41	-76.21
	Wuwei	41	38	-5.40	-11.35	-32.26	-7.23	-56.23
	Jinchang	34	32	-2.79	-5.80	-34.41	-7.41	-50.41
	Jiuquan	46	43	-5.27	-11.18	-23.32	-7.11	-46.88
	Qingyang	36	34	-2.77	0.00	-25.30	-7.35	-35.42
	Baiyin	41	40	0.00	-5.67	-8.06	0.00	-13.74
	Lanzhou	48	50	2.62	5.59	15.27	0.00	23.47
	Jiayuguan	31	32	2.84	5.89	17.92	0.00	26.65
	Gannan	37	39	2.74	5.76	25.14	7.35	40.99
	Tianshui	35	39	5.53	11.61	51.27	7.35	75.76
**Shandong**	Dongying	77	64	-20.91	-15.43	-21.01	-7.32	-64.67
	Dezhou	95	83	-19.81	-11.26	-13.57	-5.29	-49.92
	Linyi	76	67	-14.04	-11.57	-14.01	-5.49	-45.11
	Jinan	87	76	-16.95	-7.61	-13.71	-5.37	-43.64
	Laiwu	87	77	-13.56	-7.61	-13.71	-5.37	-40.25
	Jining	79	70	-13.85	-7.71	-13.93	-3.66	-39.15
	Qingdao	49	45	-7.73	-8.17	-19.26	-3.93	-39.08
	Zibo	86	76	-16.95	-7.61	-10.34	-3.60	-38.51
	Heze	91	82	-13.38	-7.56	-10.23	-5.33	-36.49
	Liaocheng	96	86	-13.20	-7.50	-10.18	-5.29	-36.17
	Zaozhuang	86	78	-13.56	-7.61	-6.89	-3.60	-31.67
	Yantai	43	40	-3.96	-4.18	-16.07	-2.00	-26.20
	Weihai	70	65	-10.68	-3.91	-7.12	-3.72	-25.43
	Weifang	36	34	-4.09	0.00	-12.84	-2.05	-18.97
	Tai'an	68	66	-3.59	-3.91	-3.58	-1.87	-12.95
	Binzhou	76	74	-3.51	-3.86	-3.50	-1.83	-12.70
	Rizhao	60	59	-3.69	0.00	-3.66	-1.90	-9.25
**Zhejiang**	Jiaxing	52	44	-12.55	-10.03	-28.09	-4.82	-55.49
	Ningbo	44	38	-12.95	-10.25	-26.34	-3.29	-52.83
	Shaoxing	53	45	-12.55	-10.03	-24.97	-4.82	-52.37
	Zhoushan	29	25	-6.97	-7.21	-30.64	-3.47	-48.28
	Taizhou	41	36	-9.86	-10.33	-23.62	-3.32	-47.14
	Lishui	38	33	-6.68	-6.99	-27.69	-3.35	-44.72
	Jinhua	53	46	-9.41	-6.69	-21.85	-3.21	-41.16
	Hangzhou	53	47	-9.41	-6.69	-15.61	-3.21	-34.92
	Wenzhou	43	39	-6.52	-3.44	-19.88	-3.29	-33.14
	Quzhou	42	42	0.00	0.00	0.00	0.00	0.00
	Huzhou	54	62	12.46	10.03	18.62	3.19	44.29
**Guangdong**	Meizhou	34	28	-8.00	-11.07	-31.10	-2.68	-52.85

	Zhuhai	31	26	-8.14	-8.43	-26.51	-2.70	-45.78
	Maoming	32	27	-8.07	-8.36	-26.15	-2.70	-45.28
	Shanwei	28	24	-8.21	-5.71	-24.41	-2.72	-41.05
	Chaozhou	38	33	-5.25	-5.49	-21.75	-2.63	-35.12
	Heyuan	34	31	-5.34	-5.53	-16.97	-1.34	-29.17
	Zhongshan	32	29	-5.38	-5.58	-14.53	-1.35	-26.83
	Shenzhen	30	27	-5.43	-2.83	-14.93	-2.70	-25.89
	Zhanjiang	28	26	-5.47	-2.85	-12.21	-1.36	-21.89
	Guangzhou	38	35	-2.62	-2.75	-13.59	-1.32	-20.28
	Yangjiang	32	30	-2.69	-5.58	-11.62	0.00	-19.89
	Shantou	32	30	-2.69	-5.58	-11.62	0.00	-19.89
	Zhaoqing	39	37	-2.60	-2.72	-8.10	-1.32	-14.75
	Shaoguan	34	33	0.00	-2.77	-5.66	0.00	-8.42
	Foshan	39	38	-2.60	-2.72	-2.70	0.00	-8.03
	Dongguan	36	35	0.00	0.00	-5.54	0.00	-5.54
	Huizhou	27	27	0.00	0.00	0.00	0.00	0.00
	Jiangmen	34	34	0.00	0.00	0.00	0.00	0.00
	Jieyang	39	39	0.00	0.00	0.00	0.00	0.00
	Qingyuan	34	35	2.67	0.00	2.83	1.34	6.83
	Yunfu	34	35	2.67	0.00	2.83	1.34	6.83
**Henan**	Xinyang	67	58	-13.35	-18.36	-54.63	-13.81	-100.15
	Nanyang	72	62	-13.16	-27.15	-45.03	-13.70	-99.05
	Zhengzhou	93	80	-18.43	-26.41	-34.71	-13.09	-92.64
	Zhoukou	79	69	-16.00	-17.98	-35.45	-13.49	-82.91
	Xuchang	78	69	-12.89	-17.98	-35.45	-13.49	-79.80
	Pingdingshan	85	75	-12.62	-26.59	-26.31	-13.29	-78.81
	Puyang	79	70	-12.80	-17.98	-35.45	-8.99	-75.22
	Sanmenxia	71	65	-9.87	-9.11	-27.02	-9.13	-55.14
	Zhumadian	71	68	-3.29	0.00	-18.01	-4.57	-25.87
	Xinxiang	89	86	-3.11	0.00	-8.72	-4.40	-16.23
	Luoyang	77	78	0.00	0.00	8.91	0.00	8.91
	Kaifeng	71	73	3.29	9.11	9.01	4.57	25.98
	Shangqiu	73	77	6.53	9.05	8.96	4.53	29.07
	Jiaozuo	83	87	6.35	8.93	17.63	4.43	37.34
	Luohe	70	77	9.87	18.23	27.17	9.13	64.40
	Hebi	66	75	13.35	18.36	45.78	13.92	91.40
	Anyang	67	87	30.04	45.90	72.84	27.61	176.39
**Fujian**	Nanping	25	22	-7.86	-8.13	-31.52	-1.96	-49.46
	Fuzhou	27	26	-3.90	-4.03	-8.74	0.00	-16.67
	Ningde	28	27	-3.86	0.00	-8.62	-1.92	-14.40
	Sanming	28	27	-3.86	0.00	-8.62	-1.92	-14.40
	Xiamen	29	28	0.00	-4.00	-8.49	0.00	-12.49
	Longyan	26	25	0.00	0.00	-8.87	-1.94	-10.81
	Putian	29	29	0.00	0.00	0.00	0.00	0.00
	Quanzhou	26	26	0.00	0.00	0.00	0.00	0.00
	Zhangzhou	33	34	0.00	3.94	8.09	0.00	12.03
**Hebei**	Langfang	83	67	-20.89	-13.30	-18.05	-7.82	-60.05
	Tangshan	82	74	-11.94	-6.65	-9.02	-3.15	-30.76
	Xingtai	95	87	-11.46	-6.51	-5.89	-3.06	-26.93
	Zhangjiakou	34	32	-3.58	-3.71	-15.16	-1.79	-24.24
	Hengshui	93	88	-5.77	-3.28	-2.96	-1.54	-13.55
	Chengde	40	39	-3.46	0.00	-7.15	-1.75	-12.36
	Handan	86	83	-5.89	-3.30	-2.99	0.00	-12.18
	Baoding	90	93	5.85	0.00	2.98	0.00	8.82
	Cangzhou	67	69	3.14	3.42	3.11	0.00	9.66
	Qinhuangdao	45	47	3.41	3.60	10.34	1.73	19.07
	Shijiazhuang	83	95	17.90	9.97	12.03	4.69	44.60
**Beijing**	Beijing	73	72	-2.62	0.00	-2.61	-1.37	-6.60
**Ningxia**	Guyuan	37	34	-4.09	-4.29	-24.96	-5.47	-38.81
	Zhongwei	46	44	-1.96	-4.16	-11.58	-5.29	-23.00
	Shizuishan	48	48	0.00	0.00	0.00	0.00	0.00
	Wuzhong	48	48	0.00	0.00	0.00	0.00	0.00
	Yinchuan	46	50	3.93	4.16	28.94	5.29	42.32
**Anhui**	Huangshan	36	28	-12.73	-33.55	-128.46	-12.73	-187.47
	Ma'anshan	60	50	-14.38	-23.54	-62.74	-15.76	-116.41
	Luan	54	46	-11.76	-15.92	-64.55	-12.01	-104.24

	Hefei	63	57	-8.56	-15.58	-31.03	-7.82	-62.98
	Suzhou	56	51	-8.75	-15.80	-31.91	-4.00	-60.47
	Tongling	55	53	-2.94	-7.90	-16.04	-4.00	-30.89
	Bengbu	63	61	-2.85	0.00	-15.51	-3.91	-22.27
	Chuzhou	61	60	0.00	-7.79	0.00	0.00	-7.79
	Bozhou	59	59	0.00	0.00	0.00	0.00	0.00
	Huaibei	58	58	0.00	0.00	0.00	0.00	0.00
	Huainan	51	53	2.99	8.02	8.16	0.00	19.17
	Wuhu	62	65	2.85	7.79	15.60	3.94	30.18
	Xuancheng	47	51	6.06	8.08	33.43	8.20	55.77
	Anqing	48	54	9.03	16.16	41.54	8.14	74.85
	Fuyang	50	62	17.91	32.07	73.89	16.27	140.15
	Chizhou	33	44	19.25	42.27	151.77	17.12	230.40
**Chongqing**	Chongqing	53	53	0.00	0.00	0.00	0.00	0.00
**Guizhou**	Anshun	28	26	-6.70	-7.01	-43.47	-8.83	-66.01
	Tongren	28	26	-6.70	-7.01	-43.47	-8.83	-66.01
	Liupanshui	42	39	-6.27	-6.64	-37.53	-8.46	-58.90
	Bijie	31	29	-3.32	-6.90	-31.47	-8.76	-50.45
	Guiyang	37	35	-3.21	-6.74	-29.43	0.00	-39.38
	Qiandongnan	30	31	0.00	6.96	21.28	0.00	28.23
	Qianxinan	20	21	3.53	7.30	24.76	0.00	35.59
	Zunyi	41	44	6.32	6.64	37.76	8.46	59.19
	Qiannan	23	30	17.33	28.72	165.22	36.29	247.56
**Tianjin**	Tianjin	68	71	3.02	0.00	6.02	1.57	10.61
**Xinjiang**	Bortala	36	29	-8.11	-12.77	-74.21	-16.17	-111.27
	Korla	22	19	-4.34	-13.49	-45.06	-11.36	-74.26
	Altay	12	10	-4.67	-14.56	-26.53	-12.01	-57.77
	Karamay	31	29	-2.08	-4.32	-19.71	-5.48	-31.60
	Hami	37	36	-2.01	-4.22	-6.14	0.00	-12.38
	Urumqi	41	40	0.00	-4.16	-5.91	0.00	-10.07
	Tarbagatay	65	70	5.42	3.92	10.40	9.93	29.67
	Hetian	95	105	6.56	3.74	9.81	13.91	34.01
	Changji	40	43	1.98	4.19	23.80	5.30	35.28
	Turpan	68	78	7.12	7.79	25.85	14.78	55.55
	Kizilsu	47	57	9.67	12.30	50.69	20.85	93.50
	Aksu	71	92	15.91	19.48	35.80	29.34	100.53
	Ili	112	155	25.06	18.32	19.31	44.73	107.42
	Kashgar	55	75	18.75	20.05	64.88	30.52	134.20
**Sichuan**	Neijiang	59	53	-8.53	-12.25	-40.90	-15.53	-77.20
	Suining	49	45	-5.90	-12.52	-42.82	-15.91	-77.16
	Nanchong	58	54	-5.68	-12.25	-24.68	-7.76	-50.38
	Dazhou	59	55	-5.68	-6.13	-24.54	-7.76	-44.11
	Liangshan	25	24	-3.28	-6.81	-21.89	0.00	-31.99
	Panzhihua	32	31	-3.17	-6.60	-19.95	0.00	-29.72
	Meishan	60	59	-2.82	0.00	-8.13	-7.70	-18.66
	Yibin	57	56	0.00	0.00	-8.23	-7.76	-15.99
	Leshan	55	54	0.00	-6.17	-8.32	0.00	-14.49
	Aba	19	19	0.00	0.00	0.00	0.00	0.00
	Chengdu	60	60	0.00	0.00	0.00	0.00	0.00
	Deyang	52	53	0.00	0.00	0.00	0.00	0.00
	Ziyang	72	74	2.72	0.00	7.87	7.52	18.11
	Guang'an	43	44	3.02	6.40	8.93	0.00	18.36
	Bazhong	34	37	6.29	6.55	38.83	8.37	60.04
	Luzhou	59	65	8.53	12.25	32.72	15.53	69.02
	Mianyang	45	50	9.00	12.71	52.95	16.18	90.82
	Zigong	39	45	9.21	12.90	74.19	16.45	112.76
	Ganzi	19	22	6.80	21.28	72.80	17.81	118.69
	Ya'an	36	43	12.49	19.65	95.16	24.89	152.18
	Guangyuan	21	26	10.11	20.94	129.16	26.47	186.68
**Jiangxi**	Shangrao	42	39	-6.47	-8.61	-36.64	-4.38	-56.11
	Jingdezhen	42	40	-3.24	-8.61	-18.32	0.00	-30.17
	Pingxiang	52	51	-3.11	-8.36	-8.52	0.00	-20.00
	Yingtan	39	39	0.00	0.00	0.00	0.00	0.00
	Jiujiang	46	47	0.00	0.00	17.77	0.00	17.77
	Fuzhou	40	41	0.00	8.68	9.28	0.00	17.95
	Ji'an	43	45	3.24	8.61	18.10	0.00	29.94

	Nanchang	40	42	3.26	8.68	18.55	0.00	30.49
	Ganzhou	41	43	3.26	0.00	27.65	4.38	35.30
	Xinyu	39	44	9.86	17.36	65.76	8.84	101.81
	Yichun	43	51	12.94	25.84	81.43	13.04	133.25
**Tibet**	Lhoka	25	24	-2.41	-5.01	-16.09	0.00	-23.51
	Nyingchi	54	54	0.00	0.00	0.00	0.00	0.00
	Shigatse	17	17	0.00	0.00	0.00	0.00	0.00
	Ngari	10	11	2.69	5.64	10.29	6.92	25.54
	Lhasa	18	20	2.52	5.21	36.27	6.54	50.55
	Naqu	24	27	4.87	10.09	49.01	6.37	70.33
	Qamdo	24	28	7.30	10.09	65.34	12.74	95.47
**Shaanxi**	Yulin	38	34	-5.60	-5.88	-50.67	-14.89	-77.05
	Yan'an	45	41	-5.43	-5.75	-39.93	-7.32	-58.42
	Shangluo	42	39	-5.47	-5.79	-32.73	-7.38	-51.38
	Ankang	47	44	-2.69	-5.71	-31.37	-7.26	-47.03
	Hanzhong	53	51	-2.63	-5.62	-7.62	0.00	-15.87
	Tongchuan	54	56	2.61	5.62	15.14	0.00	23.38
	Baoji	53	57	5.26	5.62	30.46	14.28	55.63
	Xi'an	56	71	18.14	22.33	67.37	35.42	143.26
	Weinan	56	75	23.32	27.91	82.35	42.50	176.08
	Xianyang	58	81	25.71	33.26	89.32	49.18	197.48
**Shanxi**	Changzhi	39	37	-2.93	-7.73	-25.11	-3.94	-39.71
	Taiyuan	56	56	0.00	0.00	0.00	0.00	0.00
	Yangquan	63	65	2.67	7.29	7.26	0.00	17.22
	Linfen	47	49	2.84	7.56	15.64	3.84	29.88
	Shuozhou	58	64	8.13	7.34	29.70	7.43	52.61
	Yuncheng	62	69	8.01	14.58	29.20	7.37	59.17
	Lvliang	52	58	8.32	14.90	30.38	7.55	61.16
	Datong	55	62	11.01	14.79	37.55	7.49	70.84
	Jincheng	55	63	11.01	14.79	45.05	11.24	82.10
	Xinzhou	52	62	13.87	22.35	53.17	11.33	100.72
	Jinzhong	57	73	21.85	36.98	66.82	18.58	144.24

References

1. Burnett, R.T.; Rd, P.C.; Ezzati, M.; Olives, C.; Lim, S.S.; Mehta, S.; Shin, H.H.; Singh, G.; Hubbell, B.; Brauer, M. An integrated risk function for estimating the global burden of disease attributable to ambient fine particulate matter exposure. *Environmental Health Perspectives* **2014**, *122*, 397.

© 2017 by the authors. Submitted for possible open access publication under the terms and conditions of the Creative Commons Attribution (CC BY) license (http://creativecommons.org/licenses/by/4.0/).
